# Knowledge in palliative care of nursing professionals at a Spanish hospital

**DOI:** 10.1590/1518-8345.1610.2847

**Published:** 2017-10-19

**Authors:** Elena Chover-Sierra, Antonio Martínez-Sabater, Yolanda Lapeña-Moñux

**Affiliations:** 1 Doctoral student, Facultat d’Infermeria i Podologia, Universitat de València, Valencia, Spain. Professor, Facultat d’Infermeria i Podologia, Universitat de València, Valencia, Spain. RN, Hospital General Universitario, Valencia, Spain.; 2 PhD, Professor, Facultat d’Infermeria i Podologia, Universitat de València, Valencia, Spain.; 3 PhD, Associate Professor, Faculty of Health Sciences, University Jaime I, Castellón, Spain.

**Keywords:** Nursing, Palliative Care, Education, Continuing, Education, Graduate, Education, Nursing, Education, Undergraduate

## Abstract

**Objective:**

to determine the level of knowledge in palliative care of nursing staff at a Spanish tertiary care hospital.

**Method:**

descriptive, cross-sectional study. Data were collected about the results of the Spanish version of the Palliative Care Quiz for Nurses (PCQN), sociodemographic aspects, education level and experience in the field of palliative care. Univariate and bivariate descriptive analysis was applied. Statistical significance was set at p < 0.05 in all cases.

**Results:**

159 professionals participated (mean age 39.51 years ± 10.25, with 13.96 years ± 10.79 of professional experience) 54.7% possessed experience in palliative care and 64.2% educational background (mainly basic education). The mean percentage of hits on the quiz was 54%, with statistically significant differences in function of the participants’ education and experience in palliative care.

**Conclusions:**

although the participants show sufficient knowledge on palliative care, they would benefit from a specific training program, in function of the mistaken concepts identified through the quiz, which showed to be a useful tool to diagnose professionals’ educational needs in palliative care.

## Introduction

Traditionally, cancer patients have received palliative care, although the changes in the social epidemiological patterns have entailed new indications of this care. Today, neurodegenerative conditions and organ failure in advanced stages are the most frequent non-oncologic indications^1^ ^-^ ^3^. In 2002, WHO defined palliative care as “an approach that improves the quality of life of patients and their families facing the problem associated with life-threatening illness, through the prevention and relief of suffering by means of early identification and impeccable assessment and treatment of pain and other problems, physical, psychosocial and spiritual”, further indicating the comprehensive approach, early care and quality of life as the main objectives and guidelines for its development^2^ ^-^ ^3^. 

Palliative care is considered a part of the health care systems and a fundamental element of citizens’ rights. It should be guided by the patient’s needs, considering his values, preferences, dignity and autonomy^4^ ^-^ ^5^. In Spain, these aspects have been articulated with recommendations for the basic education of all professionals and the development of specific programs that permit intervention at all care levels^4^ ^-^ ^6^. 

The European Association of Palliative Care (EAPC) proposes the development of three education levels: basic education for all nursing professionals, intermediary qualification for professionals who frequently attend to patients who need palliative care and specialized education for professionals working in specific palliative care areas^7^ ^-^ ^8^. The nursing professionals consider they are key in care practice to the population in any phase of the lifecycle, but require suitable education to deliver high-quality care. 

In Spain, undergraduate education in palliative care is included in curricula with heterogeneous contents. Similarly, at the postgraduate level, a wide range of programs exists (with difference in hours, competences and entities)^9^. 

Previous studies have analyzed nurses’ level of knowledge in palliative care and also the outcomes of educational programs in palliative care for nursing professionals, mostly without using validated tools^10^. The validated tools to measure outcomes of palliative care education include the Palliative Care Quiz for Nursing (PQCN)^11^, a self-administered questionnaire that consists of 20 multiple-choice items (true/false/does not know/did not answer) to assess three aspects of palliative care: philosophy and principles of palliative care ((4 items: 1,9, 12 and 17), control of pain and other symptoms (13 items) and psychosocial aspects of palliative care (3 items: 5, 11 and 19). According to different studies^12^ ^-^ ^22^, the PCQN has showed to be a useful tool to assess knowledge and also to identify mistaken concepts in the palliative care context. Its internal consistency according to the Cronbach test corresponds to 0.78, with correlation coefficients superior to 0.5 on before/after reliability tests undertaken as part of studies in different contexts. Its components mainly refer to aspects applicable in the clinical sphere^12^ ^,^ ^16^.

Therefore and based on the hypothesis that professionals with a background and/or experience in palliative care could score better on the quiz (larger number of hits and higher global score) than professionals without experience or education in this sphere, we intend to assess the level of knowledge of nursing staff members in the field of palliative care in a Spanish tertiary-care hospital.

## Method

Cross-sectional and descriptive study, undertaken at Hospital General Universitario de Valencia (Spain). The study population corresponded to the nursing staff working in January 2015 at the inpatient wards (360), emergency unit (80) and critical care wards (60) and who accepted to answer the questionnaire. Being a pilot study, the sample size was not delimited, although we intended to collect at least 100 questionnaires to guarantee the reliability of the results, calculating that, with this number of questionnaires and considering 60% to be the mean number of hits for the participants with an educational background in palliative care and 40% for the participants without experience, 45 subjects in each group would allow us to obtain a statistical test power superior to 70%. 

After getting approval from the Research Ethics Commission of the hospital in December 2014 and permission from Dr. Fothergill-Bourbonnais, from Ottawa University (where the original quiz was developed), the Spanish version of the PCQN was elaborated through a translation-back-translation process, previously described and used by other authors, in which two professional translators and one nursing professional who mastered both languages participated. Then, the questionnaire was reviewed by a palliative care expert committee (17) with clinical and academic experiences, aiming to analyze the content validity index (CVI) of each of the items and the global questionnaire^23^. 

The content validity analysis of the Spanish version of the PCQN showed a global content validity index (CVI) of 0.83, higher than the index considered acceptable, as CVI coefficients equal or superior to 0.78 are considered acceptable and coefficients equal or superior to 0.90 indicate high content validity^23^.

Finally, the internal consistency of the quiz was analyzed using Cronbach’s alpha coefficient, showing a coefficient of 0.67, considered acceptable by authors who defend that “the minimum acceptable reliability coefficient depends on what the tool will be used for”, making reference to the initial phases of a research and/or exploratory study^24^.

After that process, a questionnaire was designed that included, besides the PCQN translated to Spanish, a range of items to assess the participants’ sociodemographic characteristics and education and experience in palliative care. This questionnaire was distributed across the different hospitalization units that participated for the sake of the data collection, together with an information letter in which the research objectives and the anonymity and confidentiality of the data were described.

For the statistical analysis of the data, the software SPSS v.20 for Windows was applied. The questionnaire results were subject to univariate descriptive analysis, as well as the other variables used to characterize the study population. In addition, bivariate descriptive analysis was applied, using correlation studies and tests of independence among the variables, related to descriptive characteristics of the population and the questionnaire results. Parametric and non-parametric tests were used, according to the results of the normality tests. In all cases, statistical significance was set at p < 0.05.

## Results 

In total, 159 questionnaires were collected from the different participating services, which means that about 44% of the nursing professionals from the hospital participated. The questionnaires were collected as follows: Medical inpatient services 37.1%, Surgical inpatient services 25.8%, Emergencies 13.8%, Critical care services 13.2% and Maternal-infant area 10.1%, similar to the professionals’ distribution across the hospital.

### Characteristics of the population

In Table 1, the sociodemographic characteristics and aspects of the participants’ education level and experience in palliative care are displayed.

**Table 1:** Levels of experience and education in palliative care of the study population, Valencia, Spain, 2015

		Mean ± SD	N	(%)
Age		39.51 ± 10.25*		
Sex				
	Female		134	84.28
	Male		25	15.72
Years of professional experience		13.96 ± 10.79*		
Experience in PC^†^				
	Yes		87	54.7
	No		72	45.3
Experience in PC^†^ (years)		4.05 ± 4.74*		
Education in PC^†^				
	Yes		102	64.2
	No		57	35.8
Education in PC^†^ (type)				
	Higher education		32	31.4
	Continuing education		30	29.4
	University + Continuing education		24	23.5
	Postgraduate courses		6	5.9
	Postgraduate + Continuing		5	4.9
	Others		5	4.9
Education in PC^†^ (hours)				
	< 20		30	29.4
	20-50		33	32.4
	50-100		28	27.5
	> 100		11	10.8

* Mean ± Standard deviation; ^†^ PC: Palliative care

About 54.7% of the participants indicated palliative care experience (four years on average), while 64.2% indicated an educational background in that sphere, with an important percentage indicating that they gained this experience during their college education. 42.8% indicated both experience and education in this area and 24% indicated having none of both.

### Level of knowledge in palliative care

When calculating the percentage of hits (PH) and errors (PE) for each of the 20 items in the quiz, we found important variations: items 1, 4, 8 and 18 obtained the highest PH (superior to 80%) and items 5, 6, 7, 13, 17 and 19 the highest PE (superior to 40 % for items 6, 7 and 13, and superior to 60 % for items 5, 17 and 19). 

The global quiz results show a PH of 54 % (C.I. 51.93%-56.12%) and a PE of 33% (C.I. 31.48%-35.25%). The analysis of these results in details shows that 106 participants (66.7%) obtained a PH equal or superior to 50% (but only equal or superior to 65% for 39 (24.5%), and that 80 participants (50.3%) obtained a PE of 35% or higher.

In Table 2, the results are displayed for each of the subscales of the quiz: the highest PE (nearly 55%) corresponds to the psychosocial aspects and the highest PA (58.73%) to symptoms’ control. 

**Table 2:** Percentage of hits and errors on each of the subscales of the Palliative Care Quiz for Nurses (PCQN), Valencia, Spain, 2015

	Percentage Hits	C.I.*	Percentage Errors	C.I.*
Philosophy and principles	55.82% ± 24.71^†^	51.95%-59.69%	36.32% ± 23.82^†^*	32.59%-40.05%
Psychosocial aspects	31.45% ± 28.13^†^	27.04%-35.85%	54.93% ± 27.08^†^	50.68%-59.17%
Symptoms’ control	58.73% ± 16.67^†^	56.12%-61.35%	27.43% ± 15.35^†^	25.03%-29.84%

* C.I. Confidence interval of average (95%); ^†^ Mean ± standard deviation

### Variables that influence the level of knowledge in palliative care 

In Table 3, we show that both the subjects with experience and those with an educational background in palliative care obtain higher PH and lower PE. Student’s parametric t-test showed statistically significant differences between all groups, except in the PE in function of having an educational background in palliative care or not. Kruskal Wallis’ non-parametric test did not reveals significant differences in function of how many hours of education were taken (p = 0.445).

**Table 3:** Distribution of global percentage of hits and errors in function of participants’ education and experience in palliative care, Valencia, Spain, 2015

		Hits		Errors	
Experience in PC^†^					
	Yes	57.4% ± 11.8*	p < 0.001	32.1% ± 11.9*	p < 0.05
	No	49.9% ± 14.1*		34.9% ± 12.1*	
Education in PC^†^					
	Yes	55.7% ± 12.6*	p < 0.05	32.2% ± 12.6*	p = 0.11
	No	50.9% ± 14.2*		35.4% ± 10.8*	

* Mean ± standard deviation; ^†^ PC: Palliative care

In addition, the differences in the results obtained on the three quiz scales were analyzed in function of the participants’ experience and education in palliative care, using Mann Whitney’s non-parametric U-test (Table 4). Concerning the variable “experience in palliative care”, we only found statistically significant differences in the PH for the symptom’s control scale. In the analysis of the educational background in palliative care, these differences are only statistically significant for the PH on the scale philosophy and principles of palliative care.

**Table 4:** Distribution of percentage of hits and errors on each subscale of the quiz in function of participants’ education and experience in palliative care, Valencia, Spain, 2015

			Hits		Errors	
Experience in PC^†^						
	Philosophy and principles					
		Yes	57.2% ± 24.7*	p = 0.29	37.1% ± 23.8*	p = 0.70
		No	54.2% ± 24.8*		35.4% ± 24.1*	
	Psychosocial aspects					
		Yes	35.2% ± 28.9*	p = 0.07	52.5% ± 29.9*	p = 0.35
		No	26.8% ± 26.6*		57.9% ± 23.1*	
	Symptom’s control					
		Yes	62.8% ± 15.9*	p < 0.05	25.7% ± 15.8*	p = 0.11
		No	53.8% ± 16.4*		29.5% ± 14.6*	
Education in PC^†^						
	Philosophy and principles					
		Yes	58.6% ± 24.5*	p < 0.05	34.8% ± 23.4*	p = 0.25
		No	50.9% ± 24.5*		39.1% ± 24.5*	
	Psychosocial aspects					
		Yes	31.4% ± 27.8*	p = 0.96	52.9% ± 26.7*	p = 0.33
		No	31.6% ± 29.8*		58.5% ± 27.7*	
	Symptom’s control					
		Yes	60.6% ± 15.4*	p = 0.10	26.5% ± 15.6*	p = 0.22
		No	55.5% ± 18.4*		29.1% ± 14.8*	

* Mean ± standard deviation; ^†^ PC: Palliative care

The correlation between the PH and PE for the global quiz and the years of experience in palliative care was not statistically significant and showed very low Spearman’s rho (ρ) correlation coefficients (ρ = 0.071 p = 0.509 for PH, ρ = 0.010 p = 0.927 for PE).

No statistically significant correlation was found either between the years of experience in palliative care and the results of any of the three subscales: 1) philosophy and principles of palliative care ρ = -0.013, p = 0.90 for PH and ρ = 0.156, p = 0.15 for PE, 2) psychosocial aspects of palliative care ρ = 0.170, p = 0.11 for PH and ρ = -0.177, p = 0.09 for PE and 3)symptom`s control ρ = 0.037, p = 0.73 for PH and ρ = 0.013, p = 0.91 for PE).

The analysis of the correlation among the results of the different subscales, displayed in Table 5, did reveal a statistically significant correlation between the results of the subscales psychosocial aspects and philosophy and principles of palliative care, with a direct relation between the PH of both scales and an inverse relation between PH and PE.

**Table 5:** Correlation between percentages of hits and errors on the subscales of the Palliative Care Quiz for Nurses (PCQN), Valencia, Spain, 2015

		Philosophy and principles				Psychosocial aspects			Symptom’s control
		Hits	Errors		Hits	Errors		Hits	Errors
Hits philosophy and principles									
	rho		-0.78		0.27	-0.29		0.05	0.10
	p		< 0.001		< 0.001	< 0.001		0.55	0.21
Errors philosophy and principles									
	rho	-0.78			-0.19	0.26		0.09	-0.11
	p	< 0.001			< 0.05	< 0.001		0.24	0.15
Hits psychosocial aspects									
	rho	0.27	-0.19			-0.75		0.24	0.02
	p	< 0.001	< 0.05			< 0.001		0.76	0.85
Errors psychosocial aspects									
	rho	-0.29	0.26		-0.75			-0.73	0.12
	p	< 0.001	< 0.001		< 0.001			0.36	0.12
Hits symptom’s control									
	rho	0.05	0.09		0.24	-0.73			-0.76
	p	0.55	0.24		0.76	0.36			< 0.001
Errors symptom’s control									
	rho	0.10	-0.11		0.02	0.12		-0.76	
	p	0.21	0.15		0.85	0.12		< 0.001	

## Discussion

The Spanish version of the PCQN was chosen as a measure of nursing’ knowledge in palliative care because of its brevity, the possibility of self-application, because it includes questions on different areas in palliative care and because it has been translated to different languages and its distinct versions have shown its utility to measure this knowledge.

The professionals at our hospital who answered the PCQN show sufficient knowledge about palliative care, with a PH of 54%, supposing a score of 10.8 on 20. These results are only superior to the results of a study involving oncology and ICU nurses^19^, with a PH of 44.75%, and the results of nursing students in one of the validation studies of the original version of the PCQN^11^ with a PH of 46%, and highly similar to the results found in a study undertaken in Florida and involving pediatric nurses^21^, with a PH of 51.8%; and the PH obtained in the assessment of the French version of the PCQN, in which the participants also worked at different wards and very few had palliative care experience (which makes the population very similar to our study), corresponding to 54.8%^17^.

The other studies that we have reviewed presented PH bordering on or superior to 60% and involved nurses working at long-stay institutions, asylums, palliative care centers^14^ ^,^ ^16^ ^,^ ^18^ or oncology services^20^ or who had participated in palliative care training programs^12^ ^,^ ^15^ ^,^ ^22^, which would explain the better results obtained in those studies when compared to our population.

In our study, the participants with experience in palliative care presented a higher PH; although the correlation coefficients between PH and years of experience are low, and the results are not statistically significant, a direct relation between PH and years of experience in palliative care was found, as well as an inverse relation with the PE. The very low correlation coefficients between the years of experience in palliative care and the PH on the quiz can be explained by the participants’ short experience in palliative care, as most of them (84.1%) possess less than five years of experience. It could also be explained by the different characteristics of both groups (with and without palliative care experience), as the initial differences between them were not analyzed, which does not permit comparisons with greater statistical rigor.

Concerning the effect of palliative care education on the quiz results, experienced professionals present better results, although no differences were found according to the hours spent on that education. That is explained by the small size of each group and the impossibility of valid comparisons between them, in view of the lack of control for other variables, such as the number of subjects in each group and the characteristics (contents and methods) of the education programs.

On the other hand, in three of the studies reviewed^15^ ^,^ ^16^ ^,^ ^20^, the percentage of subjects with experience and education in palliative care was measured among the participants, but the effect of these variables on the quiz results was not analyzed. In another study developed in Corea in 2011^12^, the level of knowledge in palliative care was measured before the start of an education program, concluding that participants with some kind of education obtained better results at the beginning of the study.

Ronaldson et al.^19^ and Raudonis et al.^16^ measured the level of knowledge in palliative care of nursing professionals working at nursing homes, analyzing the results obtained on the three scales of the quiz. They found that the highest PH were obtained on the psychosocial aspects scale (62% and 75.67%, respectively) and the worst on the philosophy scale (50% and 57.25%, respectively), superior to the results of our study in all cases. 

Another study developed at three Iranian hospitals, involving nurses working at oncology and ICU services^13^, analyzing the results of the three subscales, found the lowest outcomes as follows: PH 37.95%, with the best PH corresponding to symptoms’ control (46.07%) and the worst to the subscale of psychosocial aspects (19.3%), justifying the low percentages found for the participants’ lack of experience and education in the field of palliative care and due to the influence of cultural and religious aspects. 

When reviewing the educational programs at our universities and studies developed in other contexts, we observe that the content is mainly focused on aspects like symptoms’ management and that the content related to the psychosocial aspects appears less frequently^10^ ^,^ ^25^. In our study, we were able to prove that the worst results correspond to the psychosocial aspects of palliative care. Therefore, we raise the need to further include these psychosocial aspects since undergraduate nursing education; thus, at least in Spain, these areas should be further addressed, not only as specific content on palliative care, but as a cross-sectional aspect throughout undergraduate and postgraduate nursing education, using participatory methods. It has been demonstrated that these methods, such as problem-based learning, clinical simulation or case studies, encourage reflection and the development of attitudes, and have been used in undergraduate and postgraduate education with positive outcomes^25^.

Concerning the most relevant items, it should be highlighted that the largest percentage of the participants answered wrongly item 5 “It is essential for the family members to stay at the patient’s side until he dies”. Hence, considering that the authors of the original questionnaire propose that the correct answer would be FALSE, in view of the many types of families the answer about the moment of confrontation with the death of a family member refers to, it is understandable that the participants in our study interpreted the question as “it is essential for some families…” (which is correct) instead of “for all families”. 

These results for item 5 are very similar to the results of studies in which the Corean version of the PCQN was sued, whose authors also justify the high percentage of wrong answers on cultural aspects of the families’ coping with death and on the fact that it is not clear whether the idem is referring to the exact moment of death or to the final days of the patient’s life(12,15). In view of the results of our study and the participants’ opinions, we proposed to reformulate item 5 as “it is essential for all families…”, to increase the probability that the Spanish professionals would answer NO, in order to maintain the correct answer by the authors of the original scale. Its elimination from the questionnaire was also raised, but discarded because that would make difficult the comparisons with other studies.

In addition, item 19, which refers to overcoming the mourning in function of the background relationship with the deceased person, presents a PE superior to 65%. This again indicates the need to further elaborate the psychological aspects of palliative care in the design of educational programs. Also, concerning that item, we identified that some participants in this pilot study indicated that they did not understood what the terms “distant or conflicting relationship” and “close or intimate relationship” referred to. This difficulty may have entailed a bias in the selection of the answer, indicating that this item should be reformulated for the sake of further clarification.

## Conclusions

The Spanish version of the PCQN showed to be a suitable instrument to rapidly assess the basic level of knowledge of nursing professionals in palliative care, in view of the brevity and self-application, despite demanding further time to manage its use and successive revisions to improve the instrument. That includes the modifications proposed in some items, with a view to obtaining a questionnaire that is more suitable to our environment.

The study results do not only offer useful information for the validation of the questionnaire, but also permit the identification of mistaken concepts and educational deficits in the field of palliative care among the nursing professionals at our hospital, as well as the existence of an average level of knowledge, which would benefit from educational activities particularly centered on the mistaken concepts identified, thus allowing the nursing professionals to offer their patients better palliative care, based on existing scientific evidence.

Nevertheless, it should be kept in mind that this study comes with a series of limitations, deriving from its concept as a pilot study of a questionnaire, undertaken at a single center. These entail the impossibility to generalize the results to any other population, of which this sample would not be representative. Therefore, we propose to continue our studies in this sense, making the proposed modifications in the questionnaire and increasing the number of participants, with a view to the generalization of the results to a larger population, such as the Spanish nursing professionals and even internationally.
